# Genetic Tools and Techniques for Recombinant Expression in Thermophilic Bacillaceae

**DOI:** 10.3390/microorganisms6020042

**Published:** 2018-05-10

**Authors:** Eivind B. Drejer, Sigrid Hakvåg, Marta Irla, Trygve Brautaset

**Affiliations:** Department of Biotechnology and Food Science, NTNU: Norwegian University of Science and Technology, 7491 Trondheim, Norway; eivind.b.drejer@ntnu.no (E.B.D.); sigrid.hakvag@ntnu.no (S.H.); marta.k.irla@ntnu.no (M.I.)

**Keywords:** recombinant expression, thermophiles, Bacillus, Geobacillus, Bacillaceae

## Abstract

Although *Escherichia coli* and *Bacillus subtilis* are the most prominent bacterial hosts for recombinant protein production by far, additional species are being explored as alternatives for production of difficult-to-express proteins. In particular, for thermostable proteins, there is a need for hosts able to properly synthesize, fold, and excrete these in high yields, and thermophilic Bacillaceae represent one potentially interesting group of microorganisms for such purposes. A number of thermophilic Bacillaceae including *B.* *methanolicus*, *B.* *coagulans*, *B.* *smithii*, *B.* *licheniformis*, *Geobacillus thermoglucosidasius*, *G. kaustophilus*, and *G. stearothermophilus* are investigated concerning physiology, genomics, genetic tools, and technologies, altogether paving the way for their utilization as hosts for recombinant production of thermostable and other difficult-to-express proteins. Moreover, recent successful deployments of CRISPR/Cas9 in several of these species have accelerated the progress in their metabolic engineering, which should increase their attractiveness for future industrial-scale production of proteins. This review describes the biology of thermophilic Bacillaceae and in particular focuses on genetic tools and methods enabling use of these organisms as hosts for recombinant protein production.

## 1. Introduction

Bacteria are widely used in science and industry as cell factories for production of recombinant proteins. The choice of bacterial host and expression system relies heavily on the origin and properties of the heterologous protein. Today, the most commonly used bacterial hosts for expression of heterologous proteins are the Gram-negative *Escherichia coli* and the Gram-positive *Bacillus subtilis*. *E. coli* is in many cases the most used host due to several advantages, including an extensively developed genetic tool box, well-known genetics and physiology, low-cost media, and rapid protein production in a short fermentation period [1]. However, this species also exhibits some limitations as the heterologous proteins are typically expressed intracellularly, which results in problems with formation of inclusion bodies and incorrect protein folding. *B. subtilis*, on the other hand, has become an industrial workhorse for recombinant protein production due to an easy cultivation, the products of its metabolism being generally recognized as safe (GRAS), ease of genetic manipulation, well-characterized expression systems, absence of significant codon bias, and exceptional ability to secrete heterologous proteins allowing cost-effective downstream processing. Lacking an outer membrane, *B. subtilis* can efficiently secrete recombinant proteins to the culture medium, therefore this bacterium is more suitable for the production of secretory recombinant proteins than *E. coli* [2,3]. Challenges in recombinant protein production in *B. subtilis* have typically been related to the activity of cell-wall-associated proteases interfering with folding of heterologous proteins in the interface between cell wall and extracellular space. This has later been ameliorated with the use of genome editing to remove the genes coding for interfering proteases [4,5,6]. Further work on *B. subtilis* to improve its properties as a host for heterologous protein production has been done, including Morimoto et al. (2008) demonstrating that a 20% reduction of genome size resulted in a proportionate increase in recombinant protein yields at the cost of lowered growth rates [7]. The status and advantages of recombinant protein production in *B. subtilis* has been reviewed thoroughly by Dijl and Hecker [8].

The successful application of *B. subtilis* as a host for the production of recombinant proteins has naturally spurred interest in exploring additional Bacillaceae for the same purpose, some of which belong to the group of thermophilic bacilli and geobacilli. Bacillaceae typically share several of the favorable traits described for *B. subtilis*. For example, most Bacillaceae species are nonpathogenic and free of exo- and endotoxins, which is particularly advantageous for production of pharmaceutical proteins [9]. Due to their cell wall composition, Bacillaceae display a remarkable ability to secrete recombinant proteins [10]. Nazina et al. (2001) reclassified several thermophilic *Bacillus* species into the new genus *Geobacillus*; in this review, we choose to treat both thermophilic *Bacillus* ssp. and *Geobacillus* ssp. as compatible systems for recombinant protein expression, and collectively refer to them as thermophilic Bacillaceae [11]. The boundary between bacterial mesophilism and thermophilism has earlier been suggested to lie in the temperature range 44–52 °C [12] and bacteria are generally classified as thermophilic if they have a temperature optimum above 50 °C [13]. Some strains of *B. coagulans* are normally cultivated below this threshold (i.e., 37 °C or 40 °C for *B. coagulans* DSM1, see Table 1), but are classified as thermophilic Bacillaceae in this review as strains with optimum growth temperatures above 50 °C also exist, e.g., *B.* coagulans 36D1 [14,15,16]. Facultative thermophiles are capable of growing at thermophilic temperatures after an adaptation phase at intermediate temperatures [12].

A general prerequisite for heterologous protein production in any microorganism is availability of functional genetic tools and gene delivery methods. Efficient protein production requires replicating plasmid vectors with constitutive and/or inducible promoters, strong ribosome binding sites, transcriptional terminators, selection markers, and functional reporter genes [17]. Genetic engineering in thermophilic bacteria presents certain specific challenges regarding stability and functionality of antibiotics, selection markers, and plasmid replication at elevated temperatures. Thermophilic Bacillaceae are currently not used for commercial production of heterologous proteins; however, their favorable physiological properties, together with current development of genetic toolboxes as presented in this review, should indicate the feasibility of these organisms as promising hosts for recombinant production in the future.

## 2. Thermophilic Bacillaceae; Evolutionary Relationship and Biotechnological Potentials

**Figure 1:**
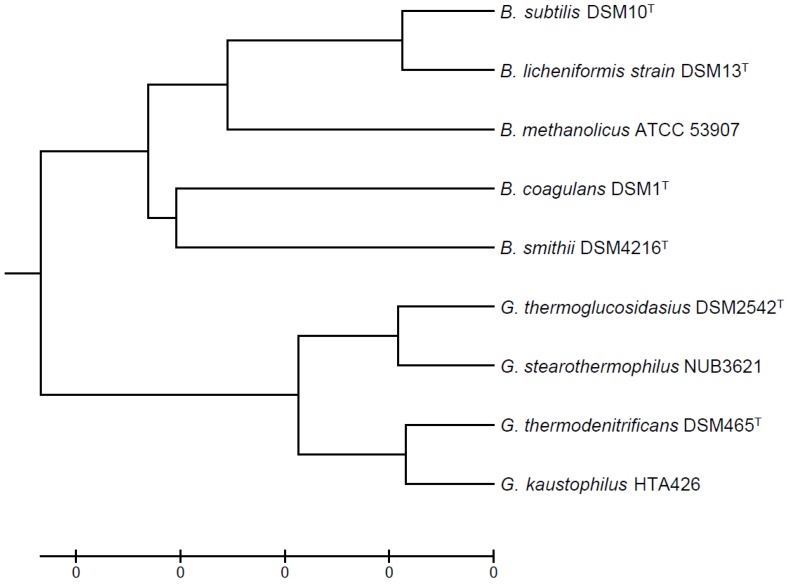
Evolutionary relationships of thermophilic Bacillaceae using *B. subtilis* as a reference strain. The evolutionary history was inferred using the UPGMA method [32]. The optimal tree with the sum of branch length = 0.22091755 is shown. The tree is drawn to scale, with branch lengths in the same units as those of the evolutionary distances used to infer the phylogenetic tree. The evolutionary distances were computed using the Maximum Composite Likelihood method [33] and are in the units of the number of base substitutions per site. The analysis involved 9 nucleotide sequences. All positions containing gaps and missing data were eliminated. There were a total of 1387 positions in the final dataset. Evolutionary analyses were conducted in MEGA7 [34].

The evolutionary relationship of thermophilic *Bacillus* and *Geobacillus* strains, selected for Table 1, is shown in Figure 1. In addition to many of the favorable properties documented for *B. subtilis*, thermophilic Bacillaceae can in addition naturally sustain growth at elevated temperatures (45–70 °C) and they can utilize a wide range of carbon sources for biomass and energy production [18,19]. One obvious advantage of looking into thermophilic Bacillaceae as hosts is exemplified in Cordova et al. where a new *Geobacillus* species, *Geobacillus* LC300, was found to grow on xylose, glucose, mannose, and galactose, with growth rates 3 times as high as that of *E. coli* on glucose and xylose [19]. Methylotrophic *B. methanolicus* grows well on both methanol and mannitol as sole carbon and energy sources at 50 °C [20,21]. Furthermore, thermophilic Bacillaceae can be used for constructing DNA libraries useful in identifying genes coding for thermostable proteins as reported by Suzuki et al. (2013) and for thermoadaptation-directed evolution of very thermolabile enzymes towards increased thermostability [22,23]. Facultatively thermophilic *B. licheniformis* is widely used in industry for secretion of native enzymes, e.g., proteases, keratinase, tannase, and others; however, most of the production experiments were performed at mesophilic temperatures [4,24,25,26,27,28,29].

Several different thermophilic Bacillaceae have been thoroughly characterized in recent years, including sequencing of their genomes and transcriptomic, proteomic, and metabolomic studies (for details, see Table 1). For most of the industrially relevant strains, data on the high cell density cultivations is also available. For example, *B. methanolicus* was cultivated to final OD_600_ = 13.5 in mannitol batch fermentation and OD_600_ = 53 in methanol fed-batch fermentation, and *G. thermoglucosidasius* (also referred to as *Parageobacillus thermoglucosidasius*) reached OD_600_ of over 10 in batch fermentation [30,31]. A representative list of controlled fermentations and high cell density experiments using thermophilic Bacillaceae, demonstrating the feasibility of high-volume cultivations of the strains, along with information on genome sequence, transcriptome, and proteome availability is presented in Table 1.

## 3. Production and Secretion of Recombinant Proteins in Thermophilic Bacillaceae

Thermophilic Bacillaceae are yet underdeveloped compared to mesophilic *B. subtilis* for recombinant protein production, although some examples are reported. Suzuki et al. (2013) were able to heterologously express the heterologous *bgaB* gene originating from *G. stearothermophilus* in *G. kaustophilus* HTA 426 host cells at 60 °C [22]. Expression of the *bgaB* gene controlled by the native inducible promoter P_gk704_ from a multicopy plasmid resulted in a final yield of 28 mg/L of the purified protein product [22]. The constructed expression system was also used to recombinantly express the PH1171c protein in yields of more than 20 mg/L in *G. kaustophilus* [22]. In addition, the expression system was able to express soluble PH0380, a protein that is insoluble when expressed in *E. coli* [22]. Suziki et al. (2013) showed that the PH1171c and AmyE proteins were produced extracellularly despite lacking fused secretion signal sequences [22]. Whether this latter result was due to cell lysis or caused by an unknown secretion mechanism remained unknown [22].

One main advantage of using Gram-positive bacteria species for recombinant expression is their general high capacity for protein secretion to the extracellular space [3]. This is accomplished using orthologous secretion systems such as the general secretion pathway (Sec pathway) and the Twin-arginine translocation pathway (Tat pathway) which use different signal peptides [72,73]. The Sec pathway is able to secrete both fully and partially unfolded proteins and is the most commonly distributed secretion system in bacteria [74]. The Tat pathway on the other hand seems complementary to the Sec pathway, commonly secreting fully folded proteins that are incompatible with Sec secretion [75]. Use of these systems for production of secreted recombinant proteins were comprehensively covered in a recent review by Freudl [76].

By far the most research on protein secretion in Bacillaceae is performed in *B. subtilis*, and limited studies have yet been reported for thermophilic Bacillaceae. Bartosiak-Jentys et al. (2013) developed a modular system for the recombinant expression and secretion of proteins in the thermophilic *G. thermoglucosidasius*, and demonstrated its application to produce a secreted heterologous endoglucanase [77]. This modular system allowed a simple mode for exchange of promoter, secretion signal peptide, and the gene of interest, enabling optimization of expression level and secretion efficiency of different heterologous proteins in *G*. *thermoglucosidasius* [77]. In a separate report signal peptide sequences of *B. subtilis* and *G. thermoglucosidasius* were investigated and surprisingly found to be similar despite the growth temperature differences in these two organisms [78]. Signal peptides have been successfully exchanged and functionally used between *B. licheniformis* to *B. subtilis*, and it is thus plausible that protein secretion in thermophilic Bacillaceae can be largely achieved by adapting well-documented functional secretion systems from mesophilic bacteria [79].

## 4. Plasmid Vectors and Transformation Methods

### 4.1. Plasmid Vectors

Plasmid vectors for thermophilic Bacillaceae presented here will focus on those that have been tested at thermophilic temperatures (above 45 °C). The range of expression vectors available for thermophilic bacilli are based on several different origins of replication derived from different plasmids: pBC1, pUB110, pMSR0, pTA1060, pBS72 (Table 2).

The most widely used plasmid vector for thermophilic bacilli is pNW33N. This shuttle plasmid contains a chloramphenicol acetyl transferase gene from *Staphylococcus aureus* plasmid pC194, an origin of replication from the *E. coli* plasmid pUC19, and the origin of replication for *Bacillus* [95]. Plasmid pNW33N has been transformed and established in several different *Bacillus* species, including *B. smithii*, *B. coagulans*, *B. licheniformis,* and *B. methanolicus* at temperatures above 45 °C [18,81,82,83,84,88]. Its derivative, pMU102, was shown to stably replicate in *Clostridium thermocellum* in temperatures up to 55 °C; temperature-sensitive variants of this plasmid were also constructed, facilitating genomic integrations in thermophilic Bacillaceae (see below) [96].

The *Staphylococcus aureus*-derived plasmid pUB110 was transformed and established in both *B. methanolicus* and *B. licheniformis* at 50 °C and 37 °C, respectively, and used for production of sfGFP in *B. methanolicus* [82,84,85]. In contrast, the pUB110-derivative pDG148Stu was not transformable to *B. coagulans* [88,97,98]. The *Bacillus*/*E. coli* shuttle vector pMSR10 is composed of the *rep* region from the native *B. coagulans* plasmid pMSR0 and ColEI region from *E. coli* plasmid pUC19 and was transformed to *B. coagulans* at 50 °C [88]. Furthermore, pTA1060-based vector pHP13 and its derivatives are routinely used for metabolic engineering of *B. methanolicus* at 50 °C, i.e., for recombinant expression of genes and gene clusters involved in biosynthesis of valued-added products such as the amino acids l-lysine and l-glutamate as well as platform chemicals cadaverine and γ-aminobutyric acid [91,92,93].

Plasmids pNW33N, pUB110, pMSR10, pHP13 all belong to the group of rolling circle plasmids, while pHCMC04 is the only theta replication plasmid that so far has been reported to be successfully transformed to any thermophilic bacilli [82,99]. It is generally assumed that theta replication plasmids are more segregationally stable than rolling circle plasmids, especially desirable for industrial fermentations that are carried out under HCDC and over many bacterial generations.

Expression vectors suitable for *Geobacillus* species were recently reviewed in Hussein et al. and Kananavičiūtė & Čitavičius [100,101]. It has been shown that popular bacilli vectors pUB110 and pNW33N can also be used in geobacilli [31,102]. Furthermore, a wide range of replicons originating from different bacteria is reported to be available for geobacilli, e.g., *Geobacillus*-derived pBST1 (theta replication type) and pSTK1 (rolling circle replication type); *Bacillus*-derived pTB19 and pTHT15. A new, versatile pBST22-based system was developed with components assembled in modular combinations to create standardized plasmids. These shuttle vectors are composed of origin of replication for geobacilli, two different antibiotic resistance markers, a promoter, a repertoire of three alternative reporter genes, and ColE1 ori for *E. coli* [87].

None of the geobacilli-derived vectors have been reported tested in thermophilic bacilli yet. There are examples of vectors with origins of replication for bacilli that are functional in geobacilli (e.g., plasmids pNW33N and pUB110); it would therefore be interesting to test if the opposite is true as well. This would mean a substantial additional contribution in vector tools availability for thermophilic bacilli and thus facilitate establishment of heterologous gene expression and protein production in these organisms.

A range of different replicons is available for thermophilic Bacillaceae, varying in copy number, type of replication, and stability. In the following chapters, further modules of expression vectors will be presented, i.e., antibiotics resistance markers, promoters, and reporter genes that function at elevated temperatures.

### 4.2. Transformation Methods

The availability of an effective transformation system is a prerequisite for establishing a genetic toolbox. Transformation methods reported to be in use for thermophilic Bacillaceae include protoplast transformation, natural competence, and electroporation [88,100,101]. Conjugative plasmid transfer has recently been described for thermophilic Bacillaceae, and electroporation is the most widely used transformation method for thermophilic strains in general [103]. The genetic accessibility of thermophilic bacilli seems to be species-specific and strain-specific [18,81]. Consequently, there is a need to continuously develop and improve the current transformation/electroporation protocols. It has been suggested that transformation of thermophilic bacteria might be impeded due to the low permeability of their plasma membranes [104].

## 5. Promoters for Regulated and Strong Expression of Recombinant Genes

Both constitutive and inducible promoters are generally necessary to ensure effective production of different heterologous proteins in bacterial hosts [17]. As presented in Table 3, the most commonly used inducible promoter in thermophilic bacilli is a xylose inducible promoter. In *B. methanolicus*, a *B. megaterium*-derived, xylose inducible promoter was demonstrated to be tightly regulated and in a dose-dependent manner [82,105]. Furthermore, this is a gratuitous promoter because *B. methanolicus* does not possess a xylose utilization pathway in its genome and thus there are no problems with inducer metabolism of the host. The native xylose inducible promoters were demonstrated to function well in *G. thermoglucosidasius, B. smithii,* and *B. licheniformis* [106,107,108]. However, all of those organisms are able to utilize xylose as a carbon source and a high basal expression from the P*_xylA_* promoter in *G. thermoglucosidasius* was observed, thus limiting its usefulness [106,107,108]. The mannitol inducible promoter, P*_mtlR_*, together with its synthetic variants, was applied in *B. methanolicus* for expression of a heterologous reporter gene, *gfpUV*; however, this promoter is not tightly regulated in *B. methanolicus* [82].

As far as constitutive promoters are concerned (Table 4), the methanol dehydrogenase gene (*mdh*) promoter has been conventionally used for strong recombinant expression of many different genes in *B. methanolicus* strain MGA3 [91,92,93]. In *B. coagulans*, the native promoters of the *pgi*, *pta*, and *ldhL* genes encoding glucose-6-phosphate isomerase, phosphotransacetylase, and lactate dehydrogenase, respectively, were successfully tested [109]. They were used to compare the effect of a *sigF* deletion on central metabolism by controlling overexpression of reporter gene *lacZ*, coding for β-galactosidase [109]. In *B. smithii*, a *B. coagulans*-derived phosphate acetyltransferase gene (*pta*) promoter was applied for expression of heterologous reporter gene and an l-lactate dehydrogenase (*ldhL*) promoter was used for expression of the native *ldhL* gene [110]. The most commonly used constitutive promoter in *B. licheniformis* is the *B. subtilis*-derived p43, which so far has solely been tested at moderate temperatures in this organism [54,111,112,113]. Interestingly, the p43 promoter is apparently stronger in *B. licheniformis* than several of its native promoters [113]. The p43 promoter was used for production of nattokinase, which is a pharmaceutical protein, in an engineered *B. licheniformis* strain deprived of extracellular protease activity [114]. The nattokinase secretion efficiency was later improved by using alternative signal peptides, which is a common engineering strategy for production of extracellular proteins [114]. The *B. subtilis*-derived surfactin operon promoter was used for controlled recombinant expression of lichenysin biosynthesis pathway genes in *B. licheniformis* at moderate temperatures [107,115].

Transcriptome data from *B. licheniformis* has been used as a basis to identify and expand the repertoire of strong promoters for high-level expression of proteins in *B. subtilis,* and this strategy allowed the identification of a promoter of similar strength to the widely used p43 promoter [50]. It would be interesting to use a similar approach for identification of constitutive promoters of varying strengths based on available transcriptome data for *B. licheniformis* and *B. methanolicus, B. coagulans* and *G. thermoglucosidasius* [30,38,49,50,70]. Furthermore, promoter libraries were screened in *G. thermoglucosidasius* in order to facilitate the fine-tuning of gene expression in this bacterium, useful for both metabolic engineering and protein production [87,108].

## 6. Antibiotics and Selection Markers Functioning at Elevated Temperatures

Antibiotic resistance genes are the most commonly used selection markers in microbial recombinant DNA technology, and functional thermostable selection markers are generally regarded as a bottleneck for genetic engineering in thermophilic microorganisms [116,117]. *E. coli*/bacilli shuttle vectors typically carry antibiotic resistance markers originating in mesophilic strains, such as chloramphenicol or kanamycin resistance genes (Cm^r^, Km^r^) from *Staphylococcus aureus* [118]. A vector carrying a tetracycline resistance marker has been successfully introduced into a moderately thermophilic strain, and the spectinomycin resistance marker was shown to work with two thermophilic strains [119]. However, the typically necessary use of low antibiotic concentrations can lead to spontaneous antibiotic-resistant colonies [120,121]. Antibiotics and resistance mechanisms used under mesophilic conditions are thus not directly applicable for work with thermophilic microorganisms due to thermal instability [122]. As an example, ampicillin has a half-life of only 3.3 h at 72 °C. However, a few antibiotics including kanamycin, neomycin, chloramphenicol, and erythromycin can be stable enough at elevated temperatures (see Table 5). Among the commonly used antibiotics in microbial molecular biology, the kanamycin molecule is reported to display the highest thermostability [122].

Mutated variants of the kanamycin resistance gene (*knt*) from *S. aureus* expressing thermostable proteins are available [123]. In *G. stearothermophilus*, a mutant Knt protein was shown to be catalytically functional at temperatures up to 70 °C, and thermostable kanamycin resistance markers have been used in development of genetic tools in different thermophilic organisms, up to 78–80 °C [124,125,126]. Directed evolution of antibiotic resistance markers of mesophilic origin resulting in isolation of thermostable mutant proteins has been reported for the antibiotics bleomycin, hygromycin, and thiostrepton [127,128,129].

Utilizing natural resistance mechanisms originating in thermophilic bacteria could circumvent problems with thermal stability of resistance markers. Production of antimicrobial compounds such as lantibiotics, other peptides, and bacteriocines by different thermophilic bacilli are well described [130,131,132,133,134]. This means that resistance mechanisms towards certain antimicrobial compounds are also then likely to exist among the thermophilic Bacillacaeae. There are, however, few reports on isolated antibiotic resistance proteins from these bacteria although production of β-lactamases for both mesophilic/thermotolerant *B. licheniformis* and a thermophilic *Bacillus* species has been reported [135,136]. Exploring this pool of genetic material is likely to result in more thermophile selection markers for use in engineering of thermophilic Bacillaceae. Early reports describe isolation of plasmids from thermophilic bacilli conferring tetracycline and kanamycin resistance on the transformed host [137,138]. The kanamycin resistance gene was later identified as the kanamycin nucleotidyltransferase gene (*knt*), similar to the gene on pUB110 from a mesophilic *Staphylococcus aureus* [139].

With the advent of metagenomics and bioprospecting of extreme environments, the discovery of new thermostable antibiotics and antibiotic resistance proteins will likely increase [140,141]. Thermophilic bacilli have been isolated from a shallow marine hot spring in Italy whereof one strain did not show sensitivity to bacitracin [142]. A thermotolerant *B. licheniformis* strain isolated from the same collection was later shown to produce an extracellular polysaccharide displaying antiviral activity [143]. Both findings should open for the unexhausted source of selection compounds from the thermophilic environments. Recently, two antibiotic resistance proteins from the Atlantis II Deep Red Sea brine pool (68 °C) were identified [144]. One of the enzymes proved to be thermostable (~40% residual activity after 30 min of incubation at 65 °C). The gene encoded a 3′-aminoglycoside phosphotransferase, and its expression resulted in both kanamycin and neomycin resistance of the host cells. Summarized, these findings should represent new strategies to obtain antibiotic resistance genes for use as selectable markers in thermophiles in general and thermophilic Bacillaceae in particular.

Both due to problems associated with low thermal stability of antibiotics and as their use in industrial scale is undesirable, auxotrophic markers could be an alternative to antibiotics for plasmid selection. Auxotrophic markers are genes essential for the biosynthesis of compounds such as uracil, tryptophan, and thymidine, which the host strain is auxotrophic for, as previously reviewed [101,117]. The system utilizing auxotrophy for uracil (due to mutations in the *pyrF* gene) has been used for *G. kaustophilus* HTA426 [145].

## 7. Reporter Proteins with Temperature Stability

Reporter proteins are important for testing, developing, and improving bacterial expression systems and for thermophilic Bacillaceae it is critical to use reporters that are functionally expressed at the elevated temperatures. For example, the widely used green fluorescent protein (GFP) has strongly reduced fluorescence activity already at 37 °C [146]. GFP mutant proteins have been constructed with increased thermal stability, such as super folder GFP and mutant super folder GFP, reported to have fluorescent activity in vivo at temperatures up to 70 °C in thermophilic Bacillaceae [81,146,147]. Additionally, Reeve et al. showed functional production of mCherry at 50 °C in *G. thermoglucosidasius* [87].

Recently, genes encoding novel flavin-binding proteins identified in genomes and metagenomics libraries of microorganisms isolated from Yellowstone National Park hot springs were characterized and expressed in vivo in the thermophilic bacterium *Thermus thermophilus*, showing activity at temperatures up to 75 °C [148]. These proteins, in addition to their stability at high temperatures, have the advantage of using flavins instead of molecular oxygen for chromophore maturation, enabling their use as reporter proteins under anaerobic conditions [148]. In addition to those thermostable reporter proteins mentioned above, the *lacZ* gene from *B. coagulans* expressing β-galactosidase as a reporter protein has been shown to work well at elevated temperatures [109]. Another β-galactosidase isolated from *G. stearothermophilus* was shown to function as a thermostable reporter in *G. thermoglucosidasius,* resulting in black colonies on plates in the presence of the lactose analogue S-gal (3,4-cyclohexenoesculetin β-d-galactopyranoside) in temperatures between 37 °C and 60 °C [149]. *G. stearothermophilus* has also been found to produce the thermostable reporter PheB, which catalyzes a reaction forming 2-hydroxymuconic semialdehyde (HMSA) [150]. This compound is reported to change the color of *G. stearothermophilus* colonies to yellow when sprayed with cathecol at 55 °C, and can alternatively be assayed spectrophotometrically in cell extracts at 375 nm [150].

## 8. Genome Editing of Thermophilic Bacillaceae

To develop bacterial species into valuable hosts for recombinant expression, the research focus must on the one side be on the expression systems and gene delivery methods as described thoroughly above, and on the other side also focus on optimization of the host cell itself, including removal of problematic proteases and methylases, improving secretion capacity, as well as other metabolic improvements. The latter focus relies much on access to tools and technologies enabling genome engineering in the relevant bacterial species. Establishing genome editing tools enabling stable integration of genetic elements into host chromosomes is especially important for industrial applications, where plasmid instability becomes problematic and industrial scale volumes of antibiotics for selection is unwanted. In principle, two alternative approaches can be taken to develop genome-editing tools for thermophilic Bacillaceae; one is by adapting mesophilic protocols to function at elevated temperatures and the second is to search for alternative genome editing tools from thermophilic sources. Several examples describe using homologous recombination as a method to knock out or replace chromosomal genes in thermophilic Bacillaceae. In 2012, Suzuki et al. developed a working *pyrF*/*pyrR* counterselection system for *G. kaustophilus*, enabling marker-free genome editing at 60 °C [145]. A similar approach was used by Kostner et al. to disrupt chromosomal alanine dehydrogenase genes in *B. licheniformis*, with the use of *codBA* as a counterselection marker at 37 °C [151]. The pGK12 plasmid, which cannot replicate at temperatures above 42 °C, was used by Wang et al. to delete the chromosomal *ldh* and *alsS* genes in *B. coagulans* [41]. After cultivation of the transformed cells at 37 °C, which is permissive for plasmid replication, a temperature shift to 50 °C caused selection for deletion mutants where the plasmid had integrated into the target chromosomal site [41]. The initial cultivation step at temperatures permissive to plasmid replication was critical, as it countered the low transformation efficiency of *B. coagulans* [41]*.* Cripps et al. used the plasmid pTMO19, a derivative of the thermosensitive pNW33N, to knock out the chromosomal *ldh* and *pflB* genes in *G. thermoglucosidasius* by using a similar strategy [31]. Bosma et al. demonstrated the use of the pNW33N plasmid to knock out the *ldhL* gene in *B. smithii* ET 138 through homologous recombination, similar to Wang et al. and Cripps et al. [110]. A counterselection marker, *lacZ*, was included on the plasmid to allow for a second crossover event and the generation of a scarless knockout mutant [110]. Interestingly, the authors reported that plasmid integration occurred without a temperature increase, which could hint at a highly efficient recombination machinery in *B. smithii* ET 138 [110]. After successful chromosomal integration was observed, the insert could be forced out in a second crossover event by applying counterselection pressure through addition of 5-bromo-4-chloro-3-indolyl-β-d-galactopyranoside (X-gal) to the medium, resulting in a scarless knockout mutant [110]. Kovacs et al. (2010) used the Cre-lox system to create knock out mutants in *B. coagulans* at elevated temperatures (45 °C) [109]. After chromosomal integration of a thermosensitive plasmid with selection markers, similar to the above-mentioned strategies, a second thermosensitive plasmid containing the Cre-lox system was transformed into the knockout mutants [109]. Cre then recombined the *lox66* and *lox71* sites flanking the *cat* selection marker on the integrated plasmid into a *lox72* site [109]. This removed the integrated plasmid, leaving behind only the *lox72* site which is not recognized by Cre [109]. The Cre-lox system could be useful in other thermophilic Bacillaceae species for establishing knockout mutants, but the requirement for functional Cre protein expression could prove challenging in other species and the scar left behind in the form of the *lox72* site limits the applications of this method. An alternative approach to using thermosensitive replicons in order to force chromosomal integration of selection markers is the use of bacterial conjugation to transfer plasmids unable to replicate in the recipient cell. In order to avoid succumbing to selection pressure, the recipient cell must integrate a selection marker flanked by homology regions into its chromosome, rendering it resistant to the selection agent. Conjugation between *E. coli* and thermophilic geobacilli was previously covered in a review by Kananavičiūtė and Čitavičius, and conjugation of a pUB110-based plasmid from *E. coli* into *B. licheniformis* was performed by Hertel et al. [101,152].

The success of the CRISPR/Cas9 system for genome editing in both prokaryotes and eukaryotes has enabled an accelerated pace in strain engineering. The most common Cas9 variant, spCas9, derived from the mesophilic bacterium *Streptococcus pyogenes* was reported to lose its catalytic activity at 42 °C [126,153]. This limits the usability of spCas9 in thermophilic Bacillaceae, but temporary cultivation temperature shifts to permissive temperatures (below 42 °C) have been shown to allow genomic modifications [126]. Mougiakos et al. demonstrated genome editing of *B. smithii* using spCas9 at 37 °C combined with homologous recombination templates [126]. Another example of genome editing by spCas9 in facultatively thermophilic bacilli was achieved by Li et al., when an spCas9 nickase mutant gene combined with homologous recombination templates was used to produce single- and multi-knock-out strains of *B. licheniformis* with high efficiencies [154]. While the strategy of lowering the growth temperature to the permissive one to allow proper enzyme function has proven to be successful, this strategy is not feasible for species that do not grow or survive in spCas9 permissive temperatures. Finding thermostable alternatives to spCas9 is therefore important for the development of a robust genome editing toolbox for thermophilic bacilli.

In 2017, Mougiakos et al. successfully applied a novel Cas9 enzyme isolated from *G. thermodenitrificans* to genetically engineer *B. smithii* [106]. This Cas9 variant was shown to retain catalytic activity in the temperature range between 20 °C and 70 °C, and with its lenient PAM sequence, this Cas9 variant should be a promising candidate for genome editing in thermophilic organisms [106].

## 9. Concluding Remarks

Thermophilic Bacillaceae typically have their growth optima around 50 °C, which on the one hand can offer process advantages with respect to thermostable protein folding and functionality, and on the other hand can present challenges with respect to availability of functional genetic tools. Today’s progress in research and development on strains belonging to this group should open for their potential use as alternative hosts for recombinant protein production at elevated temperatures. In particular, there is extensive research going on in development of genetic tools and recombinant technologies, as reviewed here, that eventually should pave the way for the use of this group of bacteria as alternative and attractive hosts for heterologous protein production. In addition, systems biology investigations to increase our understanding of their genetics and physiology, together with establishment of functional genome-editing methodologies, should open for rational improvement of strains useful hosts for recombinant expression of both proteins and biosynthetic pathways for industrial biotechnology purposes.

## Figures and Tables

**Table 1:** Overview of growth temperatures, genome sequences, accession numbers, and selected high cell density cultivations for thermophilic Bacillaceae. Explanation of abbreviations: NCBI SRA: NCBI Sequence Read Archive, PX: ProteomeXchange, Uniprot ProteomID: UPID.

Species	Strain	ΔT (°C)	T_opt_ (°C)	Sequence Data	Systems Biology Data: Accession Number (Strain)	Controlled Fermentation Conditions Main Product and Culture Volume (Strain)
*B. coagulans*	DSM 1^T^ [35]	RT/30–55 [36]	40 [35]	ALAS00000000 (whole genome shotgun sequence) [37]	Transcriptome [38] UPID: UP000031931 (DSM 1)	l-lactic acid, 2 L (C106) [39] l-lactic acid, 50 L, (DSM 23183 and DSM 23184) [40] d-lactic acid, 1.2 L and 2 L (QZ19 and D-DSM 1) [41,42],
*B. smithii*	DSM 4216^T^	25–65 [43]	55 [44]	CP012024-CP012025 (chromosome and plasmid) [44]	UPID: UP000036353 (DSM 4216), UP000011747 (7_3_47FAA)	l-lactic acid, 1 L (ET138) [18]
*B. licheniformis*	DSM 13^T^	Up to 58 [45]	51 [45]	AE017333 [46] CP000002 [47]	Genome scale model: (WX-02) [48] Transcriptome: NCBI SRA: SRP018744 (MW3Δspo, derivate of DSM 13) [49] and NCBI SRA: SRA482831 (DSM 13) [50] Proteome: PX: PXD000791 (DSM 13) [51] Metabolome [52]	Alkaline serine protease, 6 L (MW3Δspo, derivate of DSM 13) [49] *meso*-2,3-butanediol, 40 L, (MW3Δgdh) [53] *meso*-2,3-butanediol, 3 L (WX-02*ΔgdhΔacoR*) [54]
*B. methanolicus*	ATCC 53907 (MGA3)	37–65 [20]	50–53 [20]	ADWW00000000 [21]; CP007739, CP007740, and CP007741 [55]	Transcriptome: NCBI Gene Expression Omnibus, GSE64469 [30] Proteome: PX: PXD000637 and PXD000638 [56]	l-glutamate, 0.75 L (ATCC 53907) [21]
*G. thermodenitrificans*	DSM 465^T^	45–70 [57]	60 [58]	AYKT01000001.1(DSM 465) [59] CP020030-CP020032 (chromosome and plasmids) (T12) [60]	Metabolome [61] UPID: UP000001578 (NG80-2), UP000194134 (T12)	l-lactic acid, 1 L (ET 144-2) [18] l-lactic acid, 0.4 L (T12) [62]
*G. stearothermophilus*	NUB3621	39–75 [63]	67 [63]	AOTZ01000001.1 [64]	UPID: UP000037467 (ATCC 12980)	N.D
*G. kaustophilus*	JCM 12893 (HTA426)	Up to 74 [65]	60 [65]	BA000043.1 and AP006520.1 [65]	Proteome: PRIDE database, 28711–28713 [66]	N.D
*G. thermoglucosidasius*	DSM 2542^T^	40–70 [67,68]	61–63 [68]	CP012712 [67]	Genome scale model: (M10EXG) [69] and (DL33) [70] Transcriptome: (DL33) [70]	Ethanol, 1 L (NCIMB11955) [31] Pectinolytic lyases, 20 L (PB94A) [71]

**Table 2:** Plasmid vectors for thermophilic bacilli.

Origin of Replicon	Source Organism	Type of Replication in Bacilli/Geobacilli	Plasmid Name	Host Organism	Tested Temperature
pTHT15 [80]	*G. stearothermophilus*	Rolling circle	pNW33N	*B. smithii* *B. coagulans* *B. methanolicus* *B. licheniformis*	52 °C, 55 °C [18,81] 50 °C, 50 °C [18,81] 50 °C [81,82,83] 37 °C [84]
pUB110 [85]	*Staphylococcus aureus*	Rolling circle	pUB110 [85] pUB110Smp [82] pMTL61110 [86] pG2K [87]	*B. licheniformis* *B. methanolicus* *G. thermoglucosidasius* *G. thermoglucosidasius*	37 °C [84] 50 °C [82] 52 °C [86] 55 °C [87]
pMSR0 [88]	*B. coagulans*	Rolling circle	pMSR10 [88]	*B. coagulans*	50 °C [88]
pTA1060 [89]	*B. subtilis*	Rolling circle	pTH1mp [90]	*B. methanolicus*	50 °C [91,92,93]
pBS72 [94]	*B. subtilis*	Theta-replication	pBV2mp [82] pG1K [87]	*B. methanolicus* *G. thermoglucosidasius*	50 °C [82] 55 °C [87]

**Table 3:** Inducible promoters used in thermophilic Bacillaceae.

Inducer	Promoter Name	Origin of the Promoter	Host Organism	Examples of Overproduced Proteins
xylose	xpx	*B. megaterium* [105]	*B. methanolicus*	GfpUV and CadA [82]
	P*_xynA_*	*B. smithii* [106]	*B. smithii*	ThermoCas9 [106]
	P*_xyl_*	*B. licheniformis* [107]	*B. licheniformis*	Lichenysin biosynthesis pathway [107]
	P*_xynA_*	*G. thermoglucosidasius* [108]	*G. thermoglucosidasius* [108]	SfGFP [108]
mannitol	m2p	*B. methanolicus* [82]	*B. methanolicus*	GfpUV [82]

**Table 4:** Constitutive promoters used in thermophilic bacilli.

Native Gene Controlled by the Promoter	Origin of the Promoter	Host Organism	Examples of Overproduced Proteins
*mdh* encoding methanol dehydrohenase [91] *	*B. methanolicus*	*B. methanolicus* [91,92,93]	Metabolic proteins [91,92,93]
*pgi* encoding glucose-6-phosphate isomerase [109]	*B. coagulans*	*B. coagulans* [109]	LacZ [109]
*pta* encoding phosphotransacetylase [109]	*B. coagulans*	*B. coagulans* [109]	LacZ [109]
*ldhL* encoding lactate dehydrogenase [109]	*B. coagulans*	*B. coagulans* [109]	LacZ [109]
*pta* encoding phosphotransacetylase [109]	*B. coagulans*	*B. smithii* [110]	LacZ [109]
*ldhL* encoding lactate dehydrogenase [110]	*B. smithii*	*B. smithii* [110].	Lactate dehydrogenase [109]
p43 [111]	*B. subtilis*	*B. licheniformis* [54,112,113,114]	Glycerol utilization pathway [113], nattokinase [114]
*srfA* operon [115]	*B. subtilis*	*B. licheniformis*	Lichenysin biosynthesis pathway [107]
*glpFK* encoding glycerol utilization pathway [113]	*B. licheniformis*	*B. licheniformis* [113]	Glycerol utilization pathway [113]
*ytzE* encoding putative transcription factor [113]	*B. licheniformis*	*B. licheniformis* [113]	Glycerol utilization pathway [113]
*bacABC* operon coding for biosynthesis pathway of the antibiotic bacilysin [113]	*B. licheniformis*	*B. licheniformis* [113]	Glycerol utilization pathway [113]
*rplS* encoding 50S ribosomal protein L19 [87]	*G. thermoglucosidasius*	*G. thermoglucosidasius* [87]	SfGFP [87]
*groESL* operon [108]	*Geobacillus* sp. GHH01	*G. thermoglucosidasius* [108]	SfGFP [108]

* Classified as constitutive promoter due to high background expression without addition of methanol and low induction window.

**Table 5:** Half-lives of selected antibiotics incubated in anaerobic medium, 72 °C and pH 7.3 (adapted from Peteranderl et al., 1990 [122]).

Antibiotic	Half-Life (T_1/2_) (h)
Kanamycin	No detectable loss of activity
Neomycin	No detectable loss of activity
Erythromycin	77.4
Chloramphenicol	40.6
Tetracycline	5.6
Ampicillin	3.3
